# Supplementary material for: Patients’ experiences with the application of medical adhesives to the skin: a qualitative systematic review protocol
Source: BMJ Open. 2023 Jun 21;13(6):e073546. doi: 10.1136/bmjopen-2023-073546 (PMC10314666; doi:10.1136/bmjopen-2023-073546)
Supplement: Supplementary data [file bmjopen-2023-073546supp001.pdf]

**Ovid MEDLINE(R) and Epub Ahead of Print, In-Process, In-Data-Review & Other Non-Indexed Citations, Daily and Versions** January 1, 2012 to November 10, 2022
11/11/2022

| SEARCH TERMS | | | RESULTS |
|---|---|---|---|
| **Pain** | | | |
| | 1 | Pain/ or Acute Pain/ or Pain, Procedural/ or Pain Measurement/ or Pain Management/ or Pain Threshold/ | |
| | 2 | (pain* or ache* or aching or distress* or suffer* or itch* or discomfort* or anxious or anguished or agony or agonising or anxiety).ab,kf,ti. | |
| | 3 | 1 or 2 | |
| **Removal of dressings** | | | |
| | 4 | Bandages/ or Bandages, Hydrocolloid/ or Occlusive Dressings/ or Adhesives/ | |
| | 5 | Device Removal/ | |
| | 6 | (remov* or redress* or chang* or select* or application* or cho?s* or apply* or "device deficienc*" or "adverse event*").ab,kf,ti. | |
| | 7 | 5 or 6 | |
| | 8 | 4 and 7 | |
| | 9 | ((fastener* or adhesive* or tape* or taping or bandaid* or bandag* or dressing* or mucilage* or "sticky past*" or gum or latex or adherent* or adhering or seal*) adj4 (remov* or redress* or chang* or select* or application* or cho?s* or apply* or "device deficienc*" or "adverse event*")).ab,kf,ti. | |
| **Combined Sets** | | | |
| | 10 | 8 or 9 | |
| | 11 | 3 and 10 | |
| | 12 | limit 11 to (yr="2012 -Current" and (danish or dutch or english or norwegian or swedish)) | 1,703 |
