# Supplementary material for: Patients’ experiences with the application of medical adhesives to the skin: a qualitative systematic review protocol
Source: BMJ Open. 2023 Jun 21;13(6):e073546. doi: 10.1136/bmjopen-2023-073546 (PMC10314666; doi:10.1136/bmjopen-2023-073546)
Supplement: Author's manuscript [file bmjopen-2023-073546.draft_revisions.pdf]

BMJ Open is committed to open peer review. As part of this commitment we make the peer review history of every article we publish publicly available.

When an article is published we post the peer reviewers' comments and the authors' responses online. We also post the versions of the paper that were used during peer review. These are the versions that the peer review comments apply to.

The versions of the paper that follow are the versions that were submitted during the peer review process. They are not the versions of record or the final published versions. They should not be cited or distributed as the published version of this manuscript.

BMJ Open is an open access journal and the full, final, typeset and author-corrected version of record of the manuscript is available on our site with no access controls, subscription charges or pay-per-view fees (http://bmjopen.bmj.com).

If you have any questions on BMJ Open's open peer review process please email info.bmjopen@bmj.com

BMJ Open

Patients' experiences with the application of medical adhesives to the skin: a protocol for a systematic review and meta-aggregation

Journal:	*BMJ Open*
Manuscript ID	bmjopen-2023-073546
Article Type:	Protocol
Date Submitted by the Author:	09-Mar-2023
Complete List of Authors:	Hofman, Hannelore; Ghent University Faculty of Medicine and Health Sciences, University Centre for Nursing and Midwifery, Department of Public Health and Primary Care<br>Beeckman, Dimitri; Ghent University Faculty of Medicine and Health Sciences, University Centre for Nursing and Midwifery, Department of Public Health and Primary Care; Örebro University, Swedish Centre for Skin and Wound Research (SCENTR), Faculty of Medicine and Health, School of Health Sciences<br>Duljic, Tanja; Örebro University, Swedish Centre for Skin and Wound Research (SCENTR), Faculty of Medicine and Health, School of Health Sciences; Malmo University, Department of Care Science<br>Al Gilani, Samal; Dalarna University, School of Health and Welfare<br>Johansson, Sara; Creative Mammals<br>Kottner, Jan; Charite Universitatsmedizin Berlin, Institute of Clinical Nursing Science, CharitéCenter for Health and Human Sciences<br>Kinnaer, Lise-Marie; Ghent University Faculty of Medicine and Health Sciences, University Centre for Nursing and Midwifery, Department of Public Health and Primary Care<br>Eriksson, Mats; Örebro University, Faculty of Medicine and Health, School of Health Sciences
Keywords:	Systematic Review, PAIN MANAGEMENT, WOUND MANAGEMENT

SCHOLARONE™
Manuscripts

Title page

TITLE

Patients' experiences with the application of medical adhesives to the skin: a protocol for a systematic review and meta-aggregation

CORRESPONDING AUTHOR

Name: Eriksson, Mats

Address: School of Health Sciences, Faculty of Medicine and Health, School of Health Sciences, Örebro University, room P2336, Fakultetsgatan 1, SE-701 82 Örebro, Sweden

E-mail: mats.h.eriksson@oru.se

Telephone number: +46 07 7792 244

AUTHORS

Hofman, Hannelore[1]; Beeckman, Dimitri[1,2]; Duljic, Tanja[2,3]; Al Gilani, Samal[4]; Johansson, Sara[5]; Kottner, Jan[1,6]; Kinnaer, Lise-Marie[1]; Eriksson, Mats[7]

AFFILIATIONS

[1] University Centre for Nursing and Midwifery, Department of Public Health and Primary Care, Faculty of Medicine and Health Sciences, Ghent University, Ghent, Belgium

[2] Swedish Centre for Skin and Wound Research (SCENTR), Faculty of Medicine and Health, School of Health Sciences, Örebro University, Örebro, Sweden

[3] Department of Care Science, Malmö University, Malmö, Sweden

[4] School of Health and Welfare, Dalarna University, Falun, Sweden

[5] Creative Mammals, Gothenburg, Sweden

[6] Institute of Clinical Nursing Science, CharitéCenter for Health and Human Sciences, Charité-Universitätsmedizin, Berlin, Germany

[7] Faculty of Medicine and Health, School of Health Sciences, Örebro University, Örebro, Sweden

KEYWORDS

Systematic review, meta-aggregation, adhesives, skin, patient experiences, pain

WORD COUNT

1.766 words

For peer review only - http://bmjopen.bmj.com/site/about/guidelines.xhtml

Abstract

INTRODUCTION

Medical adhesives are adhesives used in medical devices to establish and maintain contact with the body over a period of time (usually by application to the skin) and are widely used in most care settings. Application of medical adhesives to the skin can lead to skin stripping, mild or severe allergic reactions and skin irritation that may manifest as redness, itching or rash. Adhesive-related skin injury can lead to infection, delayed wound healing and an increased risk of scarring. These injuries can cause severe discomfort and pain and can affect the patient's quality of life. A systematic review summarizing patient experiences on this topic will contribute to informing adhesive producers and policy makers and guiding further development and improvement of available technologies.

METHODS AND ANALYSIS

This systematic review protocol is based on the principles of the PRISMA-P guideline. A systematic search will be conducted in CINAHL, EMBASE, MEDLINE and PsycINFO. In addition, manual searches will be performed, reviewing the reference lists of relevant reviews and articles included for quality assessment. Qualitative studies using various research designs will be considered for inclusion. Screening of title, abstract and full-text will be done by two reviewers. The methodological quality of studies under consideration will be critically assessed by two reviewers using the JBI Critical Appraisal tool for Qualitative Research. Data extraction will be performed independently by two reviewers using a predefined data extraction form. Meta-aggregation will be used to summarize the empirical evidence.

ETHICS AND DISSEMINATION

No ethical approval or consent is required because no participants will be recruited. This systematic review protocol is published in an open access journal to increase transparency of the research methods used. Results will be disseminated at national and international conferences.

KEYWORDS

Systematic review, meta-aggregation, adhesives, skin, patient experiences, pain

For peer review only

Article Summary

STRENGTHS AND LIMITATIONS OF THIS STUDY

- This systematic review aims to summarize evidence on patients' experiences with the application of medical adhesives to the skin, reported in qualitative research.
- The results of this systematic review will be reported following the Enhancing Transparency in Reporting the Synthesis of Qualitative Research (ENTREQ) statement.
- The literature screening and data extraction are performed by two reviewers to minimize bias.
- The search strategy is developed in collaboration with an expert library technician and adapted to four electronic databases, relevant to this field.

Main text

INTRODUCTION

Rationale

Medical adhesives provide securement for medical devices, facilitate skin protection and healing, and allow noninvasive monitoring (1). Several definitions for medical adhesives are used in the literature (1, 2, 3, 4). In this study, medical adhesives are defined as adhesives used in medical devices to establish and maintain contact with the body over a period of time (usually by application to the skin). They are a component of a variety of health care products, including tapes, dressings, electrodes, ostomy supplies, and patches (1).

Medical adhesives are used in all patients groups in most care settings, billions of times every year (5, 6). In a cardiac-telemetry unit and a medical-surgical unit in an acute care facility in the Midwestern United States, a median number of 6.25 and 3.00 adhesive products were used per patient per day, respectively. Electrodes, peripheral intravenous (PIV) dressings, tape, surgical dressings, surgical closures, wound dressings, and peripherally inserted central catheter (PICC) dressings were the most frequently used adhesive products. The number of medical adhesive products used per patient, varied from 0 to 24 (7).

Application of medical adhesives to the skin can lead to skin stripping, mild or severe allergic reactions and skin irritation that may manifest as redness, itching or rash (1). Skin stripping injury related to the use of medical adhesives occurs when the attachment between the skin and a medical adhesive is stronger than that between the individual skin cells. This causes the epidermal layers to separate or the epidermis to detach from the dermis (1). Adhesive-related skin stripping injuries can occur at any age and in any clinical setting, but are especially prevalent in the elderly and neonates, who may have fragile skin (8, 9, 10, 11, 12). This can lead to inflammatory skin reactions, oedema and soreness, all of which can have an adverse effect on the skin barrier function (13, 14, 15). These injuries can cause severe discomfort and pain and can affect the patient's quality of life (16, 17, 18, 19). The International Association for the Study of Pain (IASP) defines pain as "an unpleasant sensory and emotional experience associated with, or resembling that associated with, actual or potential tissue damage" (20).

Some studies and guidelines on the prevention of medical adhesive-related skin injuries (MARSI) mention the risk of pain, but few authors focus on patient experiences such as pain, anxiety and discomfort (17, 21, 22, 23). The most recent review on this topic was not conducted in a systematic manner and focused on pain caused by repeated tape and dressing removal (18). Delayed wound healing due to pain, the impact on the patient's quality of life and awareness of health care practitioners were explored. However, the focus was limited to tapes and wound dressings, and did not take other types of medical adhesives into account. In addition, continuous progress is being made in the development of adhesives. Current medical adhesives adhere too strongly to the skin, resulting in medical adhesive-related skin injury (MARSI) during removal or presence on the skin (14, 24). Consequently, adhesives with sensitive removability are being developed (5, 25, 26). Due to these recent advances in adhesive research, this systematic review aims to summarize patients' experiences with the application of medical adhesives to the skin. This review will contribute to informing adhesive producers and policy makers and guiding further development and improvement of available technologies.

For peer review only - http://bmjopen.bmj.com/site/about/guidelines.xhtml

Research question

"What are the experiences of patients with the application of medical adhesives to the skin?"

METHODS

This systematic review protocol is based on the principles of the Preferred Reporting Items for Systematic review and Meta-Analysis Protocols (PRISMA-P) criteria (27). Meta-aggregation will be used to synthesize the results. Meta-aggregation is an approach that takes into account the nature and traditions of qualitative research, while also following the rigourous process of systematic review (28). This method of synthesis aims to enable generalizable recommendations to guide practioners and policy makers (29) by developing knowledge in an unbiased way, not influenced by the reviewer or outside factors (30).

Eligibility criteria

Population and context

The focus of this review will be on patients who currently or in the past have had medical adhesives applied to the skin. For the purpose of this review, there will be no restrictions on the age or sex of the population studied.

Phenomena of interest

The phenomenon of interest in this review will be the experience of patients with the application of medical adhesives to the skin.

Study design

Qualitative studies using various research designs will be eligible. Qualitative studies that describe the experience of patients with the application of medical adhesives to the skin will be considered (31).

Setting, time frame and language

There will be no restrictions on the setting. Articles with a publication date from 2012 or later will be considered. Articles published in English, Swedish, Dutch, German, Danish or Norwegian will be included in this systematic review.

Search strategy and information sources

To identify relevant studies, a two-step strategy will be used. First, a systematic search will be conducted in the following electronic databases: CINAHL (accessed through the EBSCO interface), EMBASE (accessed through Elsevier), MEDLINE (accessed through the Ovid interface) and PsycINFO (accessed through the EBSCO interface). Concepts used for the searches in MEDLINE (accessed through the Ovid interface) will be experience (keywords

For peer review only - http://bmjopen.bmj.com/site/about/guidelines.xhtml

include 'pain' and 'discomfort') and removal of dressings (keywords include 'adhesive', 'bandage', 'dressing', 'adverse event', 'device deficiency', 'removal', 'change' and 'application'). The initial search strategy, including all identified keywords and index terms, will be customized for each electronic database. Second, a manual search will be performed, reviewing the reference lists of relevant review articles and articles included for quality assessment to identify studies not covered by the search strategy.

Study selection and data collection and management

All databases will be searched individually. The results of the database searches will be exported to Covidence software for systematic reviews, which allows the recording of reasons for exclusion. Duplicates will be tracked down and subsequently removed. Literature screening will be performed by two independent reviewers. Screening will consist of two steps: (a) screening of titles and abstracts against the inclusion criteria and (b) screening of the selected full-text articles. In case of disagreement, discussions will be held until consensus is reached. The search and selection process will be summarized in a PRISMA flowchart.

Assessment of methodological quality

The methodological quality of the qualitative studies under consideration will be critically assessed by two reviewers using the Joanna Briggs Institute Critical Appraisal tool for Qualitative Research. In case of disagreement between the two reviewers, a decision will be made by consensus. If necessary, a third reviewer will be consulted.

Data extraction

Prior to database search, a data extraction form will be prepared that includes the following categories: (a) bibliographic information (lead author, year, title, journal, full citation), (b) study design and sample size, (c) population, setting and geographical context, (d) description of how the research findings are addressed in the article, (e) method of data collection, (f) method of data analysis, (g) context (product names/brands or type of material of medical adhesives investigated), (h) phenomenon of interest (experience of patients with the application of medical adhesives to the skin), and (i) findings. A finding in meta-aggregation is defined as "a verbatim extract of the author's analytical interpretation of the results or data" (31). Each extracted finding is to be accompanied by a direct quotation of the participant voice (i.e. an illustration) from the same study (32).

Data extraction will be performed independently by two reviewers. Any ambiguities will be discussed within the research team. Final data extraction will be done through discussions based on each data extraction form until consensus is reached. Quality control of the extracted data will be performed by another member of the research team on 20% of the included articles.

Data synthesis

It is expected that the search query of this study will result in a large number of qualitative studies, some of which are of poor methodological quality. Meta-aggregation will be used to summarize the empirical evidence. To each finding, the reviewer will allocate a level of

For peer review only - http://bmjopen.bmj.com/site/about/guidelines.xhtml

plausibility. Three levels of plausibility exist: unequivocal (i.e. findings accompanied by an illustration that is beyond reasonable doubt), equivocal (i.e. findings accompanied by an illustration lacking clear association with it and therefore open to challenge) and unsupported (i.e. findings that are not supported by the data). Unsupported findings will not appear in the data synthesis (32). In data synthesis, the following three steps will be conducted: (a) all findings will be extracted from the results, discussion and conclusion section of all included studies, accompanied by an illustration and will be allocated a level of plausibility (i.e. unequivocal, equivocal or unsupported), (b) findings will be summarized into categories (i.e. groups of findings) based on similarity in meaning, and (c) synthesized findings will be derived from categories (32).

The extracted findings will be presented in the text, both as a narrative summary and in a matrix consistent with the aim of this review. The report will be structured following the Enhancing Transparency in Reporting the Synthesis of Qualitative Research (ENTREQ) statement (33). The results will be synthesized and conceptualized at a higher level of abstraction to draw conclusions (32).

The relationships between the characteristics of each study and the phenomena of interest they report, as well as the relationships between the phenomena of interest of different studies, will be examined narratively by comparing and contrasting these relationships across studies. Attention will be paid to potential differences between studies, including methodological differences. Differences in patient experiences in various age groups will be examined by comparing and contrasting findings between studies for the following age groups: Newborns (0 - 1 month), Children (2 months - 11 years), Adolescents (12 - 18 years), Adults (19 - 74 years), and Elderly (75 years and older).

Patient and Public Involvement

No patients will be involved in the design or conduct of this review.

ETHICS AND DISSEMINATION

This review will use published literature and will not recruit participants. Therefore, no formal ethical approval or consent is necessary. This systematic review protocol is published in an open access journal to increase transparency of the research methods used.

This systematic review will likely provide a detailed summary of the experiences of patients with the application of medical adhesives to the skin. This review might also provide recommendations on strategies to improve patient experience with the application of medical adhesives to the skin.

The results of this systematic review will be published in a leading peer-reviewed journal in the field. Results will also be disseminated at national and international conferences.

For peer review only

Footnotes

AUTHOR CONTRIBUTIONS

All auhors contributed to the conception of the research question and the writing of the protocol. HH, DB, SJ, JK, LMK, and ME contributed to the development of search strategies, eligibility criteria, and methodology for data synthesis. All authors contributed to the draft protocol and approved the final version of this protocol. HH, TD, SAG, and ME will work in duplicate to review the titles and abstracts of all materials obtained using the search strategy to exclude articles that do not meet the eligibility criteria. HH, TD, SAG, and ME will evaluate potentially eligible studies through full-text screening and exclude non eligible studies, documenting the reason for exclusion. HH, TD, SAG, and ME will independently extract data from the included studies. HH, TD, SAG, and ME will analyze the data and draft the manuscript. All authors will read, provide feedback, and approve the final manuscript.

ACKNOWLEDGEMENTS

Mölnlycke Health Care AB provided financial support to conduct this systematic review.

FUNDING STATEMENT

This systematic review is supported by Mölnlycke Health Care AB. Mölnlycke Health Care AB is not involved in any other aspect than the funding of this systematic review. The funder will have no input on the interpretation or publication of the study results.

COMPETING INTERESTS

The authors declare that there are no competing interests.

For peer review only

For peer review only - http://bmjopen.bmj.com/site/about/guidelines.xhtml

References

1. McNichol L, Lund C, Rosen T, Gray M. Medical adhesives and patient safety: state of the science: consensus statements for the assessment, prevention, and treatment of adhesive-related skin injuries. J Wound Ostomy Continence Nurs. 2013;40(4):365-80.
2. United States Department of Health and Human Services. Medical adhesive tape and adhesive bandage [online] [cited 2023, February 2]. Available from: https://www.accessdata.fda.gov/scripts/cdrh/cfdocs/cfcfr/CFRSearch.cfm?FR=880.5240#:~:text=A%20medical%20adhesive%20tape%20or,surgical%20dressing%20without%20a%20disinfectant.
3. Widman TJ, Oostman H, Storrs FJ. Allergic contact dermatitis from medical adhesive bandages in patients who report having a reaction to medical bandages. Dermatitis. 2008;19(1):32-7.
4. Fumarola S, Allaway R, Callaghan R, Collier M, Downie F, Geraghty J, et al. Overlooked and underestimated: medical adhesive-related skin injuries. J Wound Care. 2020;29(Sup3c):S1-s24.
5. Karp JM, Langer R. Materials science: Dry solution to a sticky problem. Nature. 2011;477(7362):42-3.
6. Hadfield G, De Freitas A, Bradbury S. Clinical evaluation of a silicone adhesive remover for prevention of MARSI at dressing change. Journal of Community Nursing. 2019;33(3).
7. Farris MK, Petty M, Hamilton J, Walters SA, Flynn MA. Medical Adhesive-Related Skin Injury Prevalence Among Adult Acute Care Patients: A Single-Center Observational Study. J Wound Ostomy Continence Nurs. 2015;42(6):589-98.
8. Cutting KF. Impact of adhesive surgical tape and wound dressings on the skin, with reference to skin stripping. J Wound Care. 2008;17(4):157-8, 60-2.
9. Blaylock B, Murray M, O'Connell K, Rex J. Tape injury in the patient with total hip replacement. Orthop Nurs. 1995;14(3):25-8.
10. Noonan C, Quigley S, Curley MA. Skin integrity in hospitalized infants and children: a prevalence survey. J Pediatr Nurs. 2006;21(6):445-53.
11. McLane KM, Bookout K, McCord S, McCain J, Jefferson LS. The 2003 national pediatric pressure ulcer and skin breakdown prevalence survey: a multisite study. J Wound Ostomy Continence Nurs. 2004;31(4):168-78.
12. Jones L, Bell D, Hodgson C, Mohamud L, Stephen-Haynes J, Callaghan R, et al. Case study series: Lifteez aerosol and wipes for the prevention and management of MARSI. Wounds UK. 2018;14(5).
13. Dykes PJ, Heggie R, Hill SA. Effects of adhesive dressings on the stratum corneum of the skin. J Wound Care. 2001;10(2):7-10.
14. Cutting KF. Impact of adhesive surgical tape and wound dressings on the skin, with reference to skin stripping. J Wound Care. 2008;17(4):157-8, 60-2.
15. McNichol L, Bianchi, J. Medical adhesive-related skin injury (MARSI): made easy. London: Wounds UK. 2016.
16. Lawton S, Langøen A. Assessing and managing vulnerable periwound skin. World Wide Wounds. 2009.
17. Reevell G, Anders T, Morgan T. Improving patients' experience of dressing removal in practice. Journal of Community Nursing. 2016;30(5).
18. Collier M. Minimising pain and medical adhesive related skin injuries in vulnerable patients. Br J Nurs. 2019;28(15):S26-s32.
19. Hollinworth H, Collier M. Nurses' views about pain and trauma at dressing changes: results of a national survey. J Wound Care. 2000;9(8):369-73.
20. Raja SN, Carr DB, Cohen M, Finnerup NB, Flor H, Gibson S, et al. The revised International Association for the Study of Pain definition of pain: concepts, challenges, and compromises. Pain. 2020;161(9):1976-82.

For peer review only

21. Denyer J. Reducing pain during the removal of adhesive and adherent products. Br J Nurs. 2011;20(15):S28, s30-5.
22. Grove GL, Zerweck CR, Houser TP, Smith GE, Koski NI. A randomized and controlled comparison of gentleness of 2 medical adhesive tapes in healthy human subjects. J Wound Ostomy Continence Nurs. 2013;40(1):51-9.
23. Schaik R, Rövekamp MH. Fact or myth? Pain reduction in solvent-assisted removal of adhesive tape. J Wound Care. 2011;20(8):380-3.
24. Zulkowski K. Understanding Moisture-Associated Skin Damage, Medical Adhesive-Related Skin Injuries, and Skin Tears. Adv Skin Wound Care. 2017;30(8):372-81.
25. Moreira Lana G, Zhang X, Müller C, Hensel R, Arzt E. Film-Terminated Fibrillar Microstructures with Improved Adhesion on Skin-like Surfaces. ACS Appl Mater Interfaces. 2022;14(41):46239-51.
26. Hwang I, Kim HN, Seong M, Lee SH, Kang M, Yi H, et al. Multifunctional smart skin adhesive patches for advanced health care. Advanced healthcare materials. 2018;7(15):1800275.
27. Shamseer L, Moher D, Clarke M, Ghersi D, Liberati A, Petticrew M, et al. Preferred reporting items for systematic review and meta-analysis protocols (PRISMA-P) 2015: elaboration and explanation. Bmj. 2015;350:g7647.
28. Porritt K, Pearson A. The historical emergence of qualitative synthesis. Australia: Lippincott Williams & Wilkins; 2013.
29. Hannes K, Lockwood C. Pragmatism as the philosophical foundation for the Joanna Briggs meta-aggregative approach to qualitative evidence synthesis. J Adv Nurs. 2011;67(7):1632-42.
30. Lockwood C, Pearson A. A comparison of meta-aggregation and meta-ethnography as qualitative review methods. New York: Lippincott Williams & Wilkins; 2013.
31. The Joanna Briggs Institute. Joanna Briggs Institute Reviewers' Manual: 2014 edition. Adelaide: The Joanna Briggs Institute; 2014.
32. Lockwood C, Munn Z, Porritt K. Qualitative research synthesis: methodological guidance for systematic reviewers utilizing meta-aggregation. Int J Evid Based Healthc. 2015;13(3):179-87.
33. Tong A, Flemming K, McInnes E, Oliver S, Craig J. Enhancing transparency in reporting the synthesis of qualitative research: ENTREQ. BMC Med Res Methodol. 2012;12:181.

For peer review only

For peer review only - http://bmjopen.bmj.com/site/about/guidelines.xhtml

Reporting checklist for protocol of a systematic review and meta-analysis.

Based on the PRISMA-P guidelines.

Title: Patients' experiences with the application of medical adhesives to the skin: a protocol for a systematic review and meta-aggregation

Section and Topic		Reporting Item	Page Number and Notes
**Title**			
Identification	#1a	Identify the report as a protocol of a systematic review	Title
Update	#1b	If the protocol is for an update of a previous systematic review, identify as such	n/a: This is not an update of a previous systematic review.
**Registration**			
	#2	If registered, provide the name of the registry (such as PROSPERO) and registration number	n/a: The aim of this systematic review did not fit the scope of PROSPERO.
**Authors**			
Contact	#3a	Provide name, institutional affiliation, e-mail address of all protocol authors; provide physical mailing address of corresponding author	1
Contribution	#3b	Describe contributions of protocol authors and identify the guarantor of the review	7
**Amendments**			
	#4	If the protocol represents an amendment of a previously completed	n/a

For peer review only

		or published protocol, identify as such and list changes; otherwise, state plan for documenting important protocol amendments	
**Support**			
Sources	#5a	Indicate sources of financial or other support for the review	7
Sponsor	#5b	Provide name for the review funder and / or sponsor	7
Role of sponsor or funder	#5c	Describe roles of funder(s), sponsor(s), and / or institution(s), if any, in developing the protocol	7
**Introduction**			
Rationale	#6	Describe the rationale for the review in the context of what is already known	3
Objectives	#7	Provide an explicit statement of the question(s) the review will address with reference to participants, interventions, comparators, and outcomes (PICO)	4
**Methods**			
Eligibility criteria	#8	Specify the study characteristics (such as PICO, study design, setting, time frame) and report characteristics (such as years considered, language, publication status) to be used as criteria for eligibility for the review	4
Information sources	#9	Describe all intended information sources (such as electronic databases, contact with study authors, trial registers or other grey literature sources) with planned dates of coverage	5

For peer review only

Search strategy	#10	Present draft of search strategy to be used for at least one electronic database, including planned limits, such that it could be repeated	5
Study records - data management	#11a	Describe the mechanism(s) that will be used to manage records and data throughout the review	5
Study records - selection process	#11b	State the process that will be used for selecting studies (such as two independent reviewers) through each phase of the review (that is, screening, eligibility and inclusion in meta-analysis)	5
Study records - data collection process	#11c	Describe planned method of extracting data from reports (such as piloting forms, done independently, in duplicate), any processes for obtaining and confirming data from investigators	5
Data items	#12	List and define all variables for which data will be sought (such as PICO items, funding sources), any pre-planned data assumptions and simplifications	5
Outcomes and prioritization	#13	List and define all outcomes for which data will be sought, including prioritization of main and additional outcomes, with rationale	n/a: As this review only includes qualitative data, no outcomes can be defined. The phenomena of interest will be sought for.
Risk of bias in individual studies	#14	Describe anticipated methods for assessing risk of bias of individual studies, including whether this will be done at the outcome or study level, or both; state how this information will be used in data synthesis	5: As this study focusses on qualitative data, the methodological quality of the included studies will be assessed, not the risk of bias.

For peer review only

Data synthesis	#15a	Describe criteria under which study data will be quantitatively synthesised	n/a: As this study focusses on qualitative data, no quantitative data synthesis can take place.
Data synthesis	#15b	If data are appropriate for quantitative synthesis, describe planned summary measures, methods of handling data and methods of combining data from studies, including any planned exploration of consistency (such as I2, Kendall's τ)	n/a: As this study focusses on qualitative data, no quantitative data synthesis can take place.
Data synthesis	#15c	Describe any proposed additional analyses (such as sensitivity or subgroup analyses, meta-regression)	n/a: As this study focusses on qualitative data, no subgroup analysis or meta-regression can take place.
Data synthesis	#15d	If quantitative synthesis is not appropriate, describe the type of summary planned	6
Meta-bias(es)	#16	Specify any planned assessment of meta-bias(es) (such as publication bias across studies, selective reporting within studies)	n/a
Confidence in cumulative evidence	#17	Describe how the strength of the body of evidence will be assessed (such as GRADE)	n/a

This checklist was completed on 28. February 2023 using https://www.goodreports.org/, a tool made by the EQUATOR Network in collaboration with Penelope.ai

For peer review only

BMJ Open

Patients' experiences with the application of medical adhesives to the skin: a protocol for a systematic review and meta-aggregation

Journal:	*BMJ Open*
Manuscript ID	bmjopen-2023-073546.R1
Article Type:	Protocol
Date Submitted by the Author:	08-May-2023

Complete List of Authors:	Hofman, Hannelore; Ghent University Faculty of Medicine and Health Sciences, University Centre for Nursing and Midwifery, Department of Public Health and Primary Care<br>Beeckman, Dimitri; Ghent University Faculty of Medicine and Health Sciences, University Centre for Nursing and Midwifery, Department of Public Health and Primary Care; Örebro University, Swedish Centre for Skin and Wound Research (SCENTR), Faculty of Medicine and Health, School of Health Sciences<br>Duljic, Tanja; Örebro University, Swedish Centre for Skin and Wound Research (SCENTR), Faculty of Medicine and Health, School of Health Sciences; Malmo University, Department of Care Science<br>Al Gilani, Samal; Dalarna University, School of Health and Welfare<br>Johansson, Sara; Creative Mammals<br>Kottner, Jan; Charite Universitatsmedizin Berlin, Institute of Clinical Nursing Science, CharitéCenter for Health and Human Sciences<br>Kinnaer, Lise-Marie; Ghent University Faculty of Medicine and Health Sciences, University Centre for Nursing and Midwifery, Department of Public Health and Primary Care<br>Eriksson, Mats; Örebro University, Faculty of Medicine and Health, School of Health Sciences

**Primary Subject Heading**:	Nursing
Secondary Subject Heading:	Nursing, Qualitative research
Keywords:	Systematic Review, PAIN MANAGEMENT, WOUND MANAGEMENT

SCHOLARONE™ Manuscripts

Title page

TITLE

Patients' experiences with the application of medical adhesives to the skin: a protocol for a systematic review and meta-aggregation

CORRESPONDING AUTHOR

Name: Eriksson, Mats

Address: School of Health Sciences, Faculty of Medicine and Health, School of Health Sciences, Örebro University, room P2336, Fakultetsgatan 1, SE-701 82 Örebro, Sweden

E-mail: mats.h.eriksson@oru.se

Telephone number: +46 07 7792 244

AUTHORS

Hofman, Hannelore[1]; Beeckman, Dimitri[1,2]; Duljic, Tanja[2,3]; Al Gilani, Samal[4]; Johansson, Sara[5]; Kottner, Jan[1,6]; Kinnaer, Lise-Marie[1]; Eriksson, Mats[7]

AFFILIATIONS

[1] University Centre for Nursing and Midwifery, Department of Public Health and Primary Care, Faculty of Medicine and Health Sciences, Ghent University, Ghent, Belgium

[2] Swedish Centre for Skin and Wound Research (SCENTR), Faculty of Medicine and Health, School of Health Sciences, Örebro University, Örebro, Sweden

[3] Department of Care Science, Malmö University, Malmö, Sweden

[4] School of Health and Welfare, Dalarna University, Falun, Sweden

[5] Creative Mammals, Gothenburg, Sweden

[6] Institute of Clinical Nursing Science, CharitéCenter for Health and Human Sciences, Charité-Universitätsmedizin, Berlin, Germany

[7] Faculty of Medicine and Health, School of Health Sciences, Örebro University, Örebro, Sweden

KEYWORDS

Systematic review, meta-aggregation, adhesives, skin, patient experiences, pain

WORD COUNT

1.882 words

For peer review only - http://bmjopen.bmj.com/site/about/guidelines.xhtml

Abstract

INTRODUCTION

Medical adhesives are adhesives used in medical devices to establish and maintain contact with the body over a period of time (usually by application to the skin) and are widely used in most care settings. Application of medical adhesives to the skin can lead to skin stripping, mild or severe allergic reactions and skin irritation that may manifest as redness, itching or rash. Adhesive-related skin injury can lead to infection, delayed wound healing and an increased risk of scarring. These injuries can cause severe discomfort and pain and can affect the patient's quality of life. A systematic review summarizing patient experiences on this topic will contribute to informing adhesive producers and policy makers and guiding further development and improvement of available technologies.

METHODS AND ANALYSIS

This systematic review protocol is based on the principles of the PRISMA-P guideline. A systematic search will be conducted in CINAHL, EMBASE, MEDLINE and PsycINFO. In addition, manual searches will be performed, reviewing the reference lists of relevant reviews and articles included for quality assessment. Qualitative studies using various methods will be considered for inclusion. Screening of title, abstract and full-text will be done by two reviewers. The methodological quality of studies under consideration will be critically assessed by two reviewers using the JBI Critical Appraisal tool for Qualitative Research. Data extraction will be performed independently by two reviewers using a predefined data extraction form. Meta-aggregation will be used to summarize the evidence.

ETHICS AND DISSEMINATION

No ethical approval or consent is required because no participants will be recruited. This systematic review protocol is published in an open access journal to increase transparency of the research methods used. Results will be disseminated at national and international conferences.

KEYWORDS

Systematic review, meta-aggregation, adhesives, skin, patient experiences, pain

For peer review only

Article Summary

STRENGTHS AND LIMITATIONS OF THIS STUDY

- This systematic review aims to summarize evidence on patients' experiences with the application of medical adhesives to the skin, reported in qualitative research.
- The results of this systematic review will be reported following the Enhancing Transparency in Reporting the Synthesis of Qualitative Research (ENTREQ) statement.
- The literature screening and data extraction are performed by two reviewers to minimize bias.
- The search strategy is developed in collaboration with an expert library technician and adapted to four electronic databases, relevant to this field.

For peer review only - http://bmjopen.bmj.com/site/about/guidelines.xhtml

Main text

INTRODUCTION

Rationale

Medical adhesives provide securement for medical devices, facilitate skin protection and healing, and allow noninvasive monitoring (1). Several definitions for medical adhesives are used in the literature (1, 2, 3, 4). In this study, medical adhesives are defined as adhesives used in medical devices to establish and maintain contact with the body over a period of time (usually by application to the skin). They are a component of a variety of health care products, including tapes, dressings, electrodes, ostomy supplies, and patches (1).

Medical adhesives are used in all patients groups in most care settings, billions of times every year (5, 6). In a cardiac-telemetry unit and a medical-surgical unit in an acute care facility in the Midwestern United States, a median number of 6.25 and 3.00 adhesive products were used per patient per day, respectively. Electrodes, peripheral intravenous (PIV) dressings, tape, surgical dressings, surgical closures, wound dressings, and peripherally inserted central catheter (PICC) dressings were the most frequently used adhesive products. The number of medical adhesive products used per patient, varied from 0 to 24 (7).

Application of medical adhesives to the skin can lead to skin stripping, mild or severe allergic reactions and skin irritation that may manifest as redness, itching or rash (1). Skin stripping injury related to the use of medical adhesives occurs when the attachment between the skin and a medical adhesive is stronger than that between the individual skin cells. This causes the epidermal layers to separate or the epidermis to detach from the dermis (1). Adhesive-related skin stripping injuries can occur at any age and in any clinical setting, but are especially prevalent in the elderly and neonates, who may have fragile skin (8, 9, 10, 11, 12). This can lead to inflammatory skin reactions, oedema and soreness, all of which can have an adverse effect on the skin barrier function (13, 14, 15). These injuries can cause severe discomfort and pain and can affect the patient's quality of life (16, 17, 18, 19). The International Association for the Study of Pain (IASP) defines pain as "an unpleasant sensory and emotional experience associated with, or resembling that associated with, actual or potential tissue damage" (20).

Some studies and guidelines on the prevention of medical adhesive-related skin injuries (MARSI) mention the risk of pain, but few authors focus on patient experiences such as pain, anxiety and discomfort (17, 21, 22, 23). The most recent review on this topic was not conducted in a systematic manner and focused on pain caused by repeated tape and dressing removal (18). Delayed wound healing due to pain, the impact on the patient's quality of life and awareness of health care practitioners were explored. However, the focus was limited to tapes and wound dressings, and did not take other types of medical adhesives into account. In addition, continuous progress is being made in the development of adhesives. Current medical adhesives adhere too strongly to the skin, resulting in medical adhesive-related skin injury (MARSI) during removal or presence on the skin (14, 24). Consequently, adhesives with sensitive removability are being developed (5, 25, 26). Due to these recent advances in adhesive research, this systematic review aims to summarize patients' experiences with the application of medical adhesives to the skin. This review will contribute to informing adhesive producers and policy makers and guiding further development and improvement of available technologies.

For peer review only

For peer review only - http://bmjopen.bmj.com/site/about/guidelines.xhtml

Research question

"What are the experiences of patients with the application of medical adhesives to the skin?"

METHODS

This systematic review protocol is based on the principles of the Preferred Reporting Items for Systematic review and Meta-Analysis Protocols (PRISMA-P) criteria (27). Meta-aggregation will be used to synthesize the results. Meta-aggregation is an approach that takes into account the nature and traditions of qualitative research, while also following the rigourous process of systematic review (28). This method of synthesis aims to enable generalizable recommendations to guide practioners and policy makers (29) by developing knowledge in an unbiased way, not influenced by the reviewer or outside factors (30).

Eligibility criteria

Population and context

The focus of this review will be on patients who currently or in the past have had medical adhesives applied to the skin. For the purpose of this review, there will be no restrictions on the age or sex of the population studied.

Phenomena of interest

The phenomenon of interest in this review will be the experience of patients with the application of medical adhesives to the skin.

Study design

Qualitative studies using various methods will be eligible. Qualitative studies and qualitative data from mixed method studies that describe the experience of patients with the application of medical adhesives to the skin will be considered (31).

Setting, time frame and language

There will be no restrictions on the setting. Articles published between January 2012 and November 2022 will be considered. The search period will be restricted to the last ten years because new medical adhesives are being developed and technological advances are being made continuously (5, 24, 26). As a result, this review will focus on medical adhesives that currently are being used in clinical practice. Articles published in English, Swedish, Dutch, German, Danish or Norwegian will be included in this systematic review.

Search strategy and information sources

To identify relevant studies, a two-step strategy will be used. First, a systematic search will be conducted in the following electronic databases: CINAHL (accessed through the EBSCO

For peer review only

interface), EMBASE (accessed through Elsevier), MEDLINE (accessed through the Ovid interface) and PsycINFO (accessed through the EBSCO interface). Concepts used for the initial searches in MEDLINE (accessed through the Ovid interface) will be experience (keywords include 'pain', 'dermatitis', 'itching', 'pruritis' and 'discomfort') and removal of dressings (keywords include 'adhesive', 'bandage', 'dressing', 'adverse event', 'device deficiency', 'removal', 'change' and 'application'). The initial search strategy, including all identified keywords and index terms, will be customized for each electronic database (see supplementary file). Second, a manual search will be performed, reviewing the reference lists of relevant review articles and articles included for quality assessment to identify studies not covered by the search strategy.

Study selection and data collection and management

All databases will be searched individually. The results of the database searches will be exported to Covidence software for systematic reviews, which allows the recording of reasons for exclusion. Duplicates will be tracked down and subsequently removed. Literature screening will be performed by two independent reviewers. Screening will consist of two steps: (a) screening of titles and abstracts against the inclusion criteria and (b) screening of the selected full-text articles. In case of disagreement, discussions will be held until consensus is reached. The search and selection process will be summarized in a PRISMA flowchart.

Assessment of methodological quality

The methodological quality of the qualitative studies under consideration will be critically assessed by two reviewers using the Joanna Briggs Institute Critical Appraisal tool for Qualitative Research. In case of disagreement between the two reviewers, a decision will be made by consensus. If necessary, a third reviewer will be consulted.

Data extraction

Prior to database search, a data extraction form will be prepared that includes the following categories: (a) bibliographic information (lead author, year, title, journal, full citation), (b) study design and sample size, (c) patient demographics, medical history, setting and geographical context, (d) description of how the research findings are addressed in the article, (e) method of data collection, (f) method of data analysis, (g) context (product names/brands or type of material of medical adhesives investigated), (h) phenomenon of interest (experience of patients with the application of medical adhesives to the skin), and (i) findings. A finding in meta-aggregation is defined as "a verbatim extract of the author's analytical interpretation of the results or data" (31, 32). Each extracted finding is to be accompanied by a direct quotation of the participant voice (i.e. an illustration) from the same study (32, 33). Authors of included articles will be contacted if further clarifications concerning the conducted research are needed.

Data extraction will be performed independently by two reviewers. Any ambiguities will be discussed within the research team. Final data extraction will be done through discussions based on each data extraction form until consensus is reached. Quality control of the extracted data will be performed by another member of the research team on 20% of the included articles.

For peer review only - http://bmjopen.bmj.com/site/about/guidelines.xhtml

Data synthesis

It is expected that the search query of this study will result in a large number of studies, some of which are of poor methodological quality. Meta-aggregation will be used to summarize the evidence. To each finding, the reviewer will allocate a level of plausibility. Three levels of plausibility exist: unequivocal (i.e. findings accompanied by an illustration that is beyond reasonable doubt), equivocal (i.e. findings accompanied by an illustration lacking clear association with it and therefore open to challenge) and unsupported (i.e. findings that are not supported by the data). Unsupported findings will not appear in the data synthesis (32, 33). In meta aggregation, the following three steps will be conducted: (a) all findings will be extracted from the results, discussion and conclusion section of all included studies, accompanied by an illustration and will be allocated a level of plausibility (i.e. unequivocal, equivocal or unsupported), (b) findings will be summarized into categories (i.e. a brief description of a key concept arising from the aggregation of two or more like findings) based on similarity in meaning, and (c) synthesized findings (i.e. an overarching description of a group of categorized findings) will be derived from categories (32, 33).

The extracted findings will be presented in the text, both as a narrative summary and in a matrix consistent with the aim of this review. The report will be structured following the Enhancing Transparency in Reporting the Synthesis of Qualitative Research (ENTREQ) statement (34). The results will be synthesized and conceptualized at a higher level of abstraction to draw conclusions (32, 33).

The relationships between the characteristics of each study and the phenomena of interest they report, as well as the relationships between the phenomena of interest of different studies, will be examined narratively by comparing and contrasting these relationships across studies. Attention will be paid to potential differences between studies, including methodological differences. Differences in patient experiences in various age groups will be examined by comparing and contrasting findings between studies for the following age groups: Newborns (0 - 1 month), Children (2 months - 11 years), Adolescents (12 - 18 years), Adults (19 - 74 years), and Elderly (75 years and older).

Patient and Public Involvement

No patients will be involved in the design or conduct of this review.

ETHICS AND DISSEMINATION

This review will use published literature and will not recruit participants. Therefore, no formal ethical approval or consent is necessary. This systematic review protocol is published in an open access journal to increase transparency of the research methods used.

This systematic review will include studies that have received formal ethical approval.This systematic review will likely provide a detailed summary of the experiences of patients with the application of medical adhesives to the skin. This review might also provide recommendations on strategies to improve patient experience with the application of medical adhesives to the skin.

The results of this systematic review will be published in a leading peer-reviewed journal in the field. Results will also be disseminated at national and international conferences.

For peer review only - http://bmjopen.bmj.com/site/about/guidelines.xhtml

For peer review only

Footnotes

AUTHOR CONTRIBUTIONS

All auhors contributed to the conception of the research question and the writing of the protocol. HH, DB, SJ, JK, LMK, and ME contributed to the development of search strategies, eligibility criteria, and methodology for data synthesis. All authors contributed to the draft protocol and approved the final version of this protocol. HH, TD, SAG, and ME will work in duplicate to review the titles and abstracts of all materials obtained using the search strategy to exclude articles that do not meet the eligibility criteria. HH, TD, SAG, and ME will evaluate potentially eligible studies through full-text screening and exclude non eligible studies, documenting the reason for exclusion. HH, TD, SAG, and ME will independently extract data from the included studies. HH, TD, SAG, and ME will analyze the data and draft the manuscript. All authors will read, provide feedback, and approve the final manuscript.

ACKNOWLEDGEMENTS

Mölnlycke Health Care AB, award/grant number: NA.

FUNDING STATEMENT

Mölnlycke Health Care AB, award/grant number: NA.

Mölnlycke Health Care AB is not involved in any other aspect than the funding of this systematic review. The funder will have no input on the interpretation or publication of the study results.

COMPETING INTERESTS

The authors declare that there are no competing interests.

For peer review only

References

1. McNichol L, Lund C, Rosen T, Gray M. Medical adhesives and patient safety: state of the science: consensus statements for the assessment, prevention, and treatment of adhesive-related skin injuries. J Wound Ostomy Continence Nurs. 2013;40(4):365-80.
2. United States Department of Health and Human Services. Medical adhesive tape and adhesive bandage [online] [cited 2023, February 2]. Available from: https://www.accessdata.fda.gov/scripts/cdrh/cfdocs/cfcfr/CFRSearch.cfm?FR=880.5240#:~:text=A%20medical%20adhesive%20tape%20or,surgical%20dressing%20without%20a%20disinfectant.
3. Widman TJ, Oostman H, Storrs FJ. Allergic contact dermatitis from medical adhesive bandages in patients who report having a reaction to medical bandages. Dermatitis. 2008;19(1):32-7.
4. Fumarola S, Allaway R, Callaghan R, Collier M, Downie F, Geraghty J, et al. Overlooked and underestimated: medical adhesive-related skin injuries. J Wound Care. 2020;29(Sup3c):S1-s24.
5. Karp JM, Langer R. Materials science: Dry solution to a sticky problem. Nature. 2011;477(7362):42-3.
6. Hadfield G, De Freitas A, Bradbury S. Clinical evaluation of a silicone adhesive remover for prevention of MARSI at dressing change. Journal of Community Nursing. 2019;33(3).
7. Farris MK, Petty M, Hamilton J, Walters SA, Flynn MA. Medical Adhesive-Related Skin Injury Prevalence Among Adult Acute Care Patients: A Single-Center Observational Study. J Wound Ostomy Continence Nurs. 2015;42(6):589-98.
8. Cutting KF. Impact of adhesive surgical tape and wound dressings on the skin, with reference to skin stripping. J Wound Care. 2008;17(4):157-8, 60-2.
9. Blaylock B, Murray M, O'Connell K, Rex J. Tape injury in the patient with total hip replacement. Orthop Nurs. 1995;14(3):25-8.
10. Noonan C, Quigley S, Curley MA. Skin integrity in hospitalized infants and children: a prevalence survey. J Pediatr Nurs. 2006;21(6):445-53.
11. McLane KM, Bookout K, McCord S, McCain J, Jefferson LS. The 2003 national pediatric pressure ulcer and skin breakdown prevalence survey: a multisite study. J Wound Ostomy Continence Nurs. 2004;31(4):168-78.
12. Jones L, Bell D, Hodgson C, Mohamud L, Stephen-Haynes J, Callaghan R, et al. Case study series: Lifteez aerosol and wipes for the prevention and management of MARSI. Wounds UK. 2018;14(5).
13. Dykes PJ, Heggie R, Hill SA. Effects of adhesive dressings on the stratum corneum of the skin. J Wound Care. 2001;10(2):7-10.
14. Cutting KF. Impact of adhesive surgical tape and wound dressings on the skin, with reference to skin stripping. J Wound Care. 2008;17(4):157-8, 60-2.
15. McNichol L, Bianchi, J. Medical adhesive-related skin injury (MARSI): made easy. London: Wounds UK. 2016.
16. Lawton S, Langøen A. Assessing and managing vulnerable periwound skin. World Wide Wounds. 2009.
17. Reevell G, Anders T, Morgan T. Improving patients' experience of dressing removal in practice. Journal of Community Nursing. 2016;30(5).
18. Collier M. Minimising pain and medical adhesive related skin injuries in vulnerable patients. Br J Nurs. 2019;28(15):S26-s32.
19. Hollinworth H, Collier M. Nurses' views about pain and trauma at dressing changes: results of a national survey. J Wound Care. 2000;9(8):369-73.
20. Raja SN, Carr DB, Cohen M, Finnerup NB, Flor H, Gibson S, et al. The revised International Association for the Study of Pain definition of pain: concepts, challenges, and compromises. Pain. 2020;161(9):1976-82.

For peer review only - http://bmjopen.bmj.com/site/about/guidelines.xhtml

21. Denyer J. Reducing pain during the removal of adhesive and adherent products. Br J Nurs. 2011;20(15):S28, s30-5.
22. Grove GL, Zerweck CR, Houser TP, Smith GE, Koski NI. A randomized and controlled comparison of gentleness of 2 medical adhesive tapes in healthy human subjects. J Wound Ostomy Continence Nurs. 2013;40(1):51-9.
23. Schaik R, Rövekamp MH. Fact or myth? Pain reduction in solvent-assisted removal of adhesive tape. J Wound Care. 2011;20(8):380-3.
24. Zulkowski K. Understanding Moisture-Associated Skin Damage, Medical Adhesive-Related Skin Injuries, and Skin Tears. Adv Skin Wound Care. 2017;30(8):372-81.
25. Moreira Lana G, Zhang X, Müller C, Hensel R, Arzt E. Film-Terminated Fibrillar Microstructures with Improved Adhesion on Skin-like Surfaces. ACS Appl Mater Interfaces. 2022;14(41):46239-51.
26. Hwang I, Kim HN, Seong M, Lee SH, Kang M, Yi H, et al. Multifunctional smart skin adhesive patches for advanced health care. Advanced healthcare materials. 2018;7(15):1800275.
27. Shamseer L, Moher D, Clarke M, Ghersi D, Liberati A, Petticrew M, et al. Preferred reporting items for systematic review and meta-analysis protocols (PRISMA-P) 2015: elaboration and explanation. Bmj. 2015;350:g7647.
28. Porritt K, Pearson A. The historical emergence of qualitative synthesis. Australia: Lippincott Williams & Wilkins; 2013.
29. Hannes K, Lockwood C. Pragmatism as the philosophical foundation for the Joanna Briggs meta-aggregative approach to qualitative evidence synthesis. J Adv Nurs. 2011;67(7):1632-42.
30. Lockwood C, Pearson A. A comparison of meta-aggregation and meta-ethnography as qualitative review methods. New York: Lippincott Williams & Wilkins; 2013.
31. The Joanna Briggs Institute. Joanna Briggs Institute Reviewers' Manual: 2014 edition. Adelaide: The Joanna Briggs Institute; 2014.
32. Lockwood C, Porritt K, Munn Z, Rittenmeyer L, Salmond S, Bjerrum M, et al. Chapter 2: Systematic reviews of qualitative evidence. In: Aromataris E, Munn Z, editors. JBI Manual for Evidence Synthesis: JBI; 2020.
33. Lockwood C, Munn Z, Porritt K. Qualitative research synthesis: methodological guidance for systematic reviewers utilizing meta-aggregation. Int J Evid Based Healthc. 2015;13(3):179-87.
34. Tong A, Flemming K, McInnes E, Oliver S, Craig J. Enhancing transparency in reporting the synthesis of qualitative research: ENTREQ. BMC Med Res Methodol. 2012;12:181.

For peer review only

For peer review only - http://bmjopen.bmj.com/site/about/guidelines.xhtml

Ovid MEDLINE(R) and Epub Ahead of Print, In-Process, In-Data-Review & Other Non-Indexed Citations, Daily and Versions 1946 to November 10, 2022
11/11/2022

SEARCH TERMS			RESULTS
**Pain**			
	1	Pain/ or Acute Pain/ or Pain, Procedural/ or Pain Measurement/ or Pain Management/ or Pain Threshold/	
	2	(pain* or ache* or aching or distress* or suffer* or itch* or discomfort* or anxious or anguished or agony or agonising or anxiety).ab,kf,ti.	
	3	1 or 2	
**Removal of dressings**			
	4	Bandages/ or Bandages, Hydrocolloid/ or Occlusive Dressings/ or Adhesives/	
	5	Device Removal/	
	6	(remov* or redress* or chang* or select* or application* or cho?s* or apply* or "device deficienc*" or "adverse event*").ab,kf,ti.	
	7	5 or 6	
	8	4 and 7	
	9	((fastener* or adhesive* or tape* or taping or bandaid* or bandag* or dressing* or mucilage* or "sticky past*" or gum or latex or adherent* or adhering or seal*) adj4 (remov* or redress* or chang* or select* or application* or cho?s* or apply* or "device deficienc*" or "adverse event*")).ab,kf,ti.	
**Combined Sets**			
	10	8 or 9	
	11	3 and 10	
	12	limit 11 to (yr="2012 -Current" and (danish or dutch or english or norwegian or swedish))	1,703

For peer review only

Reporting checklist for protocol of a systematic review and meta-analysis.

Based on the PRISMA-P guidelines.

Title: Patients' experiences with the application of medical adhesives to the skin: a protocol for a systematic review and meta-aggregation

Section and Topic		Reporting Item	Page Number and Notes
**Title**			
Identification	#1a	Identify the report as a protocol of a systematic review	Title
Update	#1b	If the protocol is for an update of a previous systematic review, identify as such	n/a: This is not an update of a previous systematic review.
**Registration**			
	#2	If registered, provide the name of the registry (such as PROSPERO) and registration number	n/a: The protocol has yet to be submitted to PROSPERO.
**Authors**			
Contact	#3a	Provide name, institutional affiliation, e-mail address of all protocol authors; provide physical mailing address of corresponding author	1
Contribution	#3b	Describe contributions of protocol authors and identify the guarantor of the review	7
**Amendments**			
	#4	If the protocol represents an amendment of a previously completed or published protocol, identify as such and list changes; otherwise, state plan for documenting important protocol amendments	n/a

For peer review only

**Support**			
Sources	#5a	Indicate sources of financial or other support for the review	7
Sponsor	#5b	Provide name for the review funder and / or sponsor	7
Role of sponsor or funder	#5c	Describe roles of funder(s), sponsor(s), and / or institution(s), if any, in developing the protocol	7
**Introduction**			
Rationale	#6	Describe the rationale for the review in the context of what is already known	3
Objectives	#7	Provide an explicit statement of the question(s) the review will address with reference to participants, interventions, comparators, and outcomes (PICO)	4
**Methods**			
Eligibility criteria	#8	Specify the study characteristics (such as PICO, study design, setting, time frame) and report characteristics (such as years considered, language, publication status) to be used as criteria for eligibility for the review	4
Information sources	#9	Describe all intended information sources (such as electronic databases, contact with study authors, trial registers or other grey literature sources) with planned dates of coverage	4-5
Search strategy	#10	Present draft of search strategy to be used for at least one electronic database, including planned limits, such that it could be repeated	Supplemental Material
Study records - data management	#11a	Describe the mechanism(s) that will be used to manage records and data throughout the review	5

For peer review only

Study records - selection process	#11b	State the process that will be used for selecting studies (such as two independent reviewers) through each phase of the review (that is, screening, eligibility and inclusion in meta-analysis)	5
Study records - data collection process	#11c	Describe planned method of extracting data from reports (such as piloting forms, done independently, in duplicate), any processes for obtaining and confirming data from investigators	5
Data items	#12	List and define all variables for which data will be sought (such as PICO items, funding sources), any pre-planned data assumptions and simplifications	5
Outcomes and prioritization	#13	List and define all outcomes for which data will be sought, including prioritization of main and additional outcomes, with rationale	n/a: As this review only includes qualitative data, no outcomes can be defined. The phenomena of interest will be sought for.
Risk of bias in individual studies	#14	Describe anticipated methods for assessing risk of bias of individual studies, including whether this will be done at the outcome or study level, or both; state how this information will be used in data synthesis	5: As this study focusses on qualitative data, the methodological quality of the included studies will be assessed, not the risk of bias.
Data synthesis	#15a	Describe criteria under which study data will be quantitatively synthesised	n/a: As this study focusses on qualitative data, no quantitative data synthesis can take place.
Data synthesis	#15b	If data are appropriate for quantitative synthesis, describe planned summary measures, methods of handling data and methods of combining data from studies, including any planned exploration of consistency (such as I2, Kendall's τ)	n/a: As this study focusses on qualitative data, no quantitative data synthesis can take place.
Data synthesis	#15c	Describe any proposed additional analyses (such as sensitivity or subgroup analyses, meta-regression)	n/a: As this study focusses on qualitative data, no subgroup analysis or meta-regression can take place.

For peer review only

Data synthesis	#15d	If quantitative synthesis is not appropriate, describe the type of summary planned	6
Meta-bias(es)	#16	Specify any planned assessment of meta-bias(es) (such as publication bias across studies, selective reporting within studies)	n/a
Confidence in cumulative evidence	#17	Describe how the strength of the body of evidence will be assessed (such as GRADE)	n/a

This checklist was completed on 3. May 2023 using https://www.goodreports.org/, a tool made by the EQUATOR Network in collaboration with Penelope.ai

For peer review only

BMJ Open

Patients' experiences with the application of medical adhesives to the skin: a qualitative systematic review protocol

Journal:	*BMJ Open*
Manuscript ID	bmjopen-2023-073546.R2
Article Type:	Protocol
Date Submitted by the Author:	01-Jun-2023
Complete List of Authors:	Hofman, Hannelore; Ghent University Faculty of Medicine and Health Sciences, University Centre for Nursing and Midwifery, Department of Public Health and Primary Care<br>Beeckman, Dimitri; Ghent University Faculty of Medicine and Health Sciences, University Centre for Nursing and Midwifery, Department of Public Health and Primary Care; Örebro University, Swedish Centre for Skin and Wound Research (SCENTR), Faculty of Medicine and Health, School of Health Sciences<br>Duljic, Tanja; Örebro University, Swedish Centre for Skin and Wound Research (SCENTR), Faculty of Medicine and Health, School of Health Sciences; Malmo University, Department of Care Science<br>Al Gilani, Samal; Dalarna University, School of Health and Welfare<br>Johansson, Sara; Creative Mammals<br>Kottner, Jan; Charite Universitatsmedizin Berlin, Institute of Clinical Nursing Science, CharitéCenter for Health and Human Sciences<br>Kinnaer, Lise-Marie; Ghent University Faculty of Medicine and Health Sciences, University Centre for Nursing and Midwifery, Department of Public Health and Primary Care<br>Eriksson, Mats; Örebro University, Faculty of Medicine and Health, School of Health Sciences
**Primary Subject Heading**:	Nursing
Secondary Subject Heading:	Nursing, Qualitative research
Keywords:	Systematic Review, PAIN MANAGEMENT, WOUND MANAGEMENT

SCHOLARONE™
Manuscripts

Title page

TITLE

Patients' experiences with the application of medical adhesives to the skin: a qualitative systematic review protocol

CORRESPONDING AUTHOR

Name: Eriksson, Mats

Address: School of Health Sciences, Faculty of Medicine and Health, School of Health Sciences, Örebro University, room P2336, Fakultetsgatan 1, SE-701 82 Örebro, Sweden

E-mail: mats.h.eriksson@oru.se

Telephone number: +46 07 7792 244

AUTHORS

Hofman, Hannelore[1]; Beeckman, Dimitri[1,2]; Duljic, Tanja[2,3]; Al Gilani, Samal[4]; Johansson, Sara[5]; Kottner, Jan[1,6]; Kinnaer, Lise-Marie[1]; Eriksson, Mats[7]

AFFILIATIONS

[1] University Centre for Nursing and Midwifery, Department of Public Health and Primary Care, Faculty of Medicine and Health Sciences, Ghent University, Ghent, Belgium

[2] Swedish Centre for Skin and Wound Research (SCENTR), Faculty of Medicine and Health, School of Health Sciences, Örebro University, Örebro, Sweden

[3] Department of Care Science, Malmö University, Malmö, Sweden

[4] School of Health and Welfare, Dalarna University, Falun, Sweden

[5] Creative Mammals, Gothenburg, Sweden

[6] Institute of Clinical Nursing Science, CharitéCenter for Health and Human Sciences, Charité-Universitätsmedizin, Berlin, Germany

[7] Faculty of Medicine and Health, School of Health Sciences, Örebro University, Örebro, Sweden

KEYWORDS

Systematic review, meta-aggregation, adhesives, skin, patient experiences, pain

WORD COUNT

1.892 words

For peer review only - http://bmjopen.bmj.com/site/about/guidelines.xhtml

Abstract

INTRODUCTION

Medical adhesives are adhesives used in medical devices to establish and maintain contact with the body over a period of time (usually by application to the skin) and are widely used in most care settings. Application of medical adhesives to the skin can lead to skin stripping, mild or severe allergic reactions and skin irritation that may manifest as redness, itching or rash. Adhesive-related skin injury can lead to infection, delayed wound healing and an increased risk of scarring. These injuries can cause severe discomfort and pain and can affect the patient's quality of life. A systematic review summarizing patient experiences on this topic will contribute to informing adhesive producers and policy makers and guiding further development and improvement of available technologies.

METHODS AND ANALYSIS

This systematic review protocol is based on the principles of the PRISMA-P guideline. A systematic search will be conducted in CINAHL, EMBASE, MEDLINE and PsycINFO. In addition, manual searches will be performed, reviewing the reference lists of relevant reviews and articles included for quality assessment. Qualitative studies using various methods will be considered for inclusion. Screening of title, abstract and full-text will be done by two reviewers. The methodological quality of studies under consideration will be critically assessed by two reviewers using the JBI Critical Appraisal tool for Qualitative Research. Data extraction will be performed independently by two reviewers using a predefined data extraction form. Meta-aggregation will be used to summarize the evidence.

ETHICS AND DISSEMINATION

No ethical approval or consent is required because no participants will be recruited. This systematic review protocol is published in an open access journal to increase transparency of the research methods used. Results will be disseminated at national and international conferences.

KEYWORDS

Systematic review, meta-aggregation, adhesives, skin, patient experiences, pain

For peer review only

Article Summary

STRENGTHS AND LIMITATIONS OF THIS STUDY

- The results of this systematic review will be reported following the Enhancing Transparency in Reporting the Synthesis of Qualitative Research (ENTREQ) statement.
- The literature screening, quality assessment and data extraction will be performed by two reviewers to minimize bias.
- The search strategy is developed in collaboration with an expert library technician and adapted to four electronic databases, relevant to this field.
- This systematic review will only include articles published in English, Swedish, Dutch, German, Norwegian or Danish, which may introduce language bias.

For peer review only - http://bmjopen.bmj.com/site/about/guidelines.xhtml

Main text

INTRODUCTION

Rationale

Medical adhesives provide securement for medical devices, facilitate skin protection and healing, and allow noninvasive monitoring (1). Several definitions for medical adhesives are used in the literature (1, 2, 3). In this study, medical adhesives are defined as adhesives used in medical devices to establish and maintain contact with the body over a period of time (usually by application to the skin). They are a component of a variety of health care products, including tapes, dressings, electrodes, ostomy supplies, and patches (1).

Medical adhesives are used in all patients groups in most care settings, billions of times every year (4, 5). In a cardiac-telemetry unit and a medical-surgical unit in an acute care facility in the Midwestern United States, a median number of 6.25 and 3.00 adhesive products were used per patient per day, respectively. Electrodes, peripheral intravenous (PIV) dressings, tape, surgical dressings, surgical closures, wound dressings, and peripherally inserted central catheter (PICC) dressings were the most frequently used adhesive products. The number of medical adhesive products used per patient, varied from 0 to 24 (6).

Application of medical adhesives to the skin can lead to skin stripping, mild or severe allergic reactions and skin irritation that may manifest as redness, itching or rash (1). Skin stripping injury related to the use of medical adhesives occurs when the attachment between the skin and a medical adhesive is stronger than that between the individual skin cells. This causes the epidermal layers to separate or the epidermis to detach from the dermis (1). Adhesive-related skin stripping injuries can occur at any age and in any clinical setting, but are especially prevalent in the elderly and neonates, who may have fragile skin (7, 8, 9). This can lead to inflammatory skin reactions, oedema and soreness, all of which can have an adverse effect on the skin barrier function (10, 11, 12). These injuries can cause severe discomfort and pain and can affect the patient's quality of life (13, 14, 15, 16). The International Association for the Study of Pain (IASP) defines pain as "an unpleasant sensory and emotional experience associated with, or resembling that associated with, actual or potential tissue damage" (17).

Some studies and guidelines on the prevention of medical adhesive-related skin injuries (MARSI) mention the risk of pain, but few authors focus on patient experiences such as pain, anxiety and discomfort (13, 18, 19, 20). The most recent review on this topic was not conducted in a systematic manner and focused on pain caused by repeated tape and dressing removal (14). Delayed wound healing due to pain, the impact on the patient's quality of life and awareness of health care practitioners were explored. However, the focus was limited to tapes and wound dressings, and did not take other types of medical adhesives into account. In addition, continuous progress is being made in the development of adhesives. Current medical adhesives adhere too strongly to the skin, resulting in medical adhesive-related skin injury (MARSI) during removal or presence on the skin (10, 21). Consequently, adhesives with sensitive removability are being developed (4, 22, 23). Due to these recent advances in adhesive research, this systematic review aims to summarize patients' experiences with the application of medical adhesives to the skin. This review will contribute to informing adhesive producers and policy makers and guiding further development and improvement of available technologies.

Research question

"What are the experiences of patients with the application of medical adhesives to the skin?"

For peer review only - http://bmjopen.bmj.com/site/about/guidelines.xhtml

METHODS

This systematic review protocol is based on the principles of the Preferred Reporting Items for Systematic review and Meta-Analysis Protocols (PRISMA-P) criteria (24). Meta-aggregation will be used to synthesize the results. Meta-aggregation is an approach that takes into account the nature and traditions of qualitative research, while also following the rigourous process of systematic review (25). This method of synthesis aims to enable generalizable recommendations to guide practioners and policy makers (26) by developing knowledge in an unbiased way, not influenced by the reviewer or outside factors (27).

Eligibility criteria

Population and context

The focus of this review will be on patients who currently or in the past have had medical adhesives applied to the skin. For the purpose of this review, there will be no restrictions on the age or sex of the population studied.

Phenomena of interest

The phenomenon of interest in this review will be the experience of patients with the application of medical adhesives to the skin.

Study design

Qualitative studies using various methods will be eligible. Qualitative studies and qualitative data from mixed method studies that describe the experience of patients with the application of medical adhesives to the skin will be considered (28).

Setting, time frame and language

There will be no restrictions on the setting. Articles published between January 2012 and November 2022 will be considered. The search period will be restricted to the last ten years because new medical adhesives are being developed and technological advances are being made continuously (4, 21, 23). As a result, this review will focus on medical adhesives that currently are being used in clinical practice. Articles published in languages in which at least two members of the review team are proficient, namely English, Swedish, Dutch, German, Danish and Norwegian, will be included in this systematic review.

Search strategy and information sources

To identify relevant studies, a two-step strategy will be used. First, a systematic search will be conducted in the following electronic databases: CINAHL (accessed through the EBSCO interface), EMBASE (accessed through Elsevier), MEDLINE (accessed through the Ovid interface) and PsycINFO (accessed through the EBSCO interface). Concepts used for the initial searches in MEDLINE (accessed through the Ovid interface) will be experience (keywords include ‘pain’, ‘dermatitis’, ‘itching’, ‘pruritis’ and ‘discomfort’) and removal of

For peer review only - http://bmjopen.bmj.com/site/about/guidelines.xhtml

dressings (keywords include 'adhesive', 'bandage', 'dressing', 'adverse event', 'device deficiency', 'removal', 'change' and 'application'). The initial search strategy, including all identified keywords and index terms, will be customized for each electronic database (see supplementary file). Second, a manual search will be performed, reviewing the reference lists of relevant review articles and articles included for quality assessment to identify studies not covered by the search strategy.

Study selection and data collection and management

All databases will be searched individually. The results of the database searches will be exported to Covidence software for systematic reviews, which allows the recording of reasons for exclusion. Duplicates will be tracked down and subsequently removed. Literature screening will be performed by two independent reviewers. Screening will consist of two steps: (a) screening of titles and abstracts against the inclusion criteria and (b) screening of the selected full-text articles. In case of disagreement, discussions will be held until consensus is reached. The search and selection process will be summarized in a PRISMA flowchart.

Assessment of methodological quality

The methodological quality of the qualitative studies under consideration will be critically assessed by two reviewers using the Joanna Briggs Institute Critical Appraisal tool for Qualitative Research. In case of disagreement between the two reviewers, a decision will be made by consensus. If necessary, a third reviewer will be consulted.

Data extraction

Prior to database search, a data extraction form will be prepared that includes the following categories: (a) bibliographic information (lead author, year, title, journal, full citation), (b) study design and sample size, (c) patient demographics, medical history, setting and geographical context, (d) description of how the research findings are addressed in the article, (e) method of data collection, (f) method of data analysis, (g) context (product names/brands or type of material of medical adhesives investigated), (h) phenomenon of interest (experience of patients with the application of medical adhesives to the skin), and (i) findings. A finding in meta-aggregation is defined as "a verbatim extract of the author's analytical interpretation of the results or data" (28). Each extracted finding is to be accompanied by a direct quotation of the participant voice (i.e. an illustration) from the same study (28, 29). Authors of included articles will be contacted if further clarifications concerning the conducted research are needed.

Data extraction will be performed independently by two reviewers. Any ambiguities will be discussed within the research team. Final data extraction will be done through discussions based on each data extraction form until consensus is reached. Quality control of the extracted data will be performed by another member of the research team on 20% of the included articles.

For peer review only - http://bmjopen.bmj.com/site/about/guidelines.xhtml

Data synthesis

It is expected that the search query of this study will result in a large number of studies, some of which are of poor methodological quality. Meta-aggregation will be used to summarize the evidence. To each finding, the reviewer will allocate a level of plausibility. Three levels of plausibility exist: unequivocal (i.e. findings accompanied by an illustration that is beyond reasonable doubt), equivocal (i.e. findings accompanied by an illustration lacking clear association with it and therefore open to challenge) and unsupported (i.e. findings that are not supported by the data). Unsupported findings will not appear in the data synthesis (28, 29). In meta aggregation, the following three steps will be conducted: (a) all findings will be extracted from the results, discussion and conclusion section of all included studies, accompanied by an illustration and will be allocated a level of plausibility (i.e. unequivocal, equivocal or unsupported), (b) findings will be summarized into categories (i.e. a brief description of a key concept arising from the aggregation of two or more like findings) based on similarity in meaning, and (c) synthesized findings (i.e. an overarching description of a group of categorized findings) will be derived from categories (28, 29).

The extracted findings will be presented in the text, both as a narrative summary and in a matrix consistent with the aim of this review. The report will be structured following the Enhancing Transparency in Reporting the Synthesis of Qualitative Research (ENTREQ) statement (30). The results will be synthesized and conceptualized at a higher level of abstraction to draw conclusions (28, 29).

The relationships between the characteristics of each study and the phenomena of interest they report, as well as the relationships between the phenomena of interest of different studies, will be examined narratively by comparing and contrasting these relationships across studies. Attention will be paid to potential differences between studies, including methodological differences. Differences in patient experiences in various age groups will be examined by comparing and contrasting findings between studies for the following age groups: Newborns (0 - 1 month), Children (2 months - 11 years), Adolescents (12 - 18 years), Adults (19 - 74 years), and Elderly (75 years and older).

Patient and Public Involvement

No patients will be involved in the design or conduct of this review.

ETHICS AND DISSEMINATION

This review will use published literature and will not recruit participants. Therefore, no formal ethical approval or consent is necessary. This systematic review protocol is published in an open access journal to increase transparency of the research methods used.

This systematic review will include studies that have received formal ethical approval.This systematic review will likely provide a detailed summary of the experiences of patients with the application of medical adhesives to the skin. This review might also provide recommendations on strategies to improve patient experience with the application of medical adhesives to the skin.

The results of this systematic review will be published in a leading peer-reviewed journal in the field. Results will also be disseminated at national and international conferences.

For peer review only - http://bmjopen.bmj.com/site/about/guidelines.xhtml

Footnotes

AUTHOR CONTRIBUTIONS

All auhors contributed to the conception of the research question and the writing of the protocol. HH, DB, SJ, JK, LMK, and ME contributed to the development of search strategies, eligibility criteria, and methodology for data synthesis. All authors contributed to the draft protocol and approved the final version of this protocol. HH, TD, SAG, and ME will work in duplicate to review the titles and abstracts of all materials obtained using the search strategy to exclude articles that do not meet the eligibility criteria. HH, TD, SAG, and ME will evaluate potentially eligible studies through full-text screening and exclude non eligible studies, documenting the reason for exclusion. HH, TD, SAG, and ME will independently extract data from the included studies. HH, TD, SAG, and ME will analyze the data and draft the manuscript. All authors will read, provide feedback, and approve the final manuscript.

ACKNOWLEDGEMENTS

Mölnlycke Health Care AB, award/grant number: NA.

FUNDING STATEMENT

Mölnlycke Health Care AB, award/grant number: NA.

Mölnlycke Health Care AB is not involved in any other aspect than the funding of this systematic review. The funder will have no input on the interpretation or publication of the study results.

COMPETING INTERESTS

The authors declare that there are no competing interests.

For peer review only

References

1. McNichol L, Lund C, Rosen T, Gray M. Medical adhesives and patient safety: state of the science: consensus statements for the assessment, prevention, and treatment of adhesive-related skin injuries. J Wound Ostomy Continence Nurs. 2013;40(4):365-80.
2. United States Department of Health and Human Services. Medical adhesive tape and adhesive bandage [online] [cited 2023, February 2]. Available from: https://www.accessdata.fda.gov/scripts/cdrh/cfdocs/cfcfr/CFRSearch.cfm?FR=880.5240#:~:text=A%20medical%20adhesive%20tape%20or,surgical%20dressing%20without%20a%20disinfectant.
3. Fumarola S, Allaway R, Callaghan R, Collier M, Downie F, Geraghty J, et al. Overlooked and underestimated: medical adhesive-related skin injuries. J Wound Care. 2020;29(Sup3c):S1-s24.
4. Karp JM, Langer R. Materials science: Dry solution to a sticky problem. Nature. 2011;477(7362):42-3.
5. Hadfield G, De Freitas A, Bradbury S. Clinical evaluation of a silicone adhesive remover for prevention of MARSI at dressing change. Journal of Community Nursing. 2019;33(3).
6. Farris MK, Petty M, Hamilton J, Walters SA, Flynn MA. Medical Adhesive-Related Skin Injury Prevalence Among Adult Acute Care Patients: A Single-Center Observational Study. J Wound Ostomy Continence Nurs. 2015;42(6):589-98.
7. Cutting KF. Impact of adhesive surgical tape and wound dressings on the skin, with reference to skin stripping. J Wound Care. 2008;17(4):157-8, 60-2.
8. Jones L, Bell D, Hodgson C, Mohamud L, Stephen-Haynes J, Callaghan R, et al. Case study series: Lifteez aerosol and wipes for the prevention and management of MARSI. Wounds UK. 2018;14(5).
9. August DL, New K, Ray RA, Kandasamy Y. Frequency, location and risk factors of neonatal skin injuries from mechanical forces of pressure, friction, shear and stripping: a systematic literature review. Journal of Neonatal Nursing. 2018;24(4):173-80.
10. Cutting KF. Impact of adhesive surgical tape and wound dressings on the skin, with reference to skin stripping. J Wound Care. 2008;17(4):157-8, 60-2.
11. McNichol L, Bianchi, J. Medical adhesive-related skin injury (MARSI): made easy. London: Wounds UK. 2016.
12. Matsumura H, Imai R, Ahmatjan N, Ida Y, Gondo M, Shibata D, et al. Removal of adhesive wound dressing and its effects on the stratum corneum of the skin: comparison of eight different adhesive wound dressings. Int Wound J. 2014;11(1):50-4.
13. Reevell G, Anders T, Morgan T. Improving patients' experience of dressing removal in practice. Journal of Community Nursing. 2016;30(5).
14. Collier M. Minimising pain and medical adhesive related skin injuries in vulnerable patients. Br J Nurs. 2019;28(15):S26-s32.
15. Kim JY, Kim NK, Lee YJ. A descriptive study of Korean nurses' perception of pain and skin tearing at dressing change. Int Wound J. 2016;13 Suppl 1(Suppl 1):47-51.
16. Bianchi J. Protecting the integrity of the periwound skin. Wound Essentials. 2012;1:58-64.
17. Raja SN, Carr DB, Cohen M, Finnerup NB, Flor H, Gibson S, et al. The revised International Association for the Study of Pain definition of pain: concepts, challenges, and compromises. Pain. 2020;161(9):1976-82.
18. Denyer J. Reducing pain during the removal of adhesive and adherent products. Br J Nurs. 2011;20(15):S28, s30-5.
19. Grove GL, Zerweck CR, Houser TP, Smith GE, Koski NI. A randomized and controlled comparison of gentleness of 2 medical adhesive tapes in healthy human subjects. J Wound Ostomy Continence Nurs. 2013;40(1):51-9.

For peer review only - http://bmjopen.bmj.com/site/about/guidelines.xhtml

20. Schaik R, Rövekamp MH. Fact or myth? Pain reduction in solvent-assisted removal of adhesive tape. J Wound Care. 2011;20(8):380-3.
21. Zulkowski K. Understanding Moisture-Associated Skin Damage, Medical Adhesive-Related Skin Injuries, and Skin Tears. Adv Skin Wound Care. 2017;30(8):372-81.
22. Moreira Lana G, Zhang X, Müller C, Hensel R, Arzt E. Film-Terminated Fibrillar Microstructures with Improved Adhesion on Skin-like Surfaces. ACS Appl Mater Interfaces. 2022;14(41):46239-51.
23. Hwang I, Kim HN, Seong M, Lee SH, Kang M, Yi H, et al. Multifunctional smart skin adhesive patches for advanced health care. Advanced healthcare materials. 2018;7(15):1800275.
24. Shamseer L, Moher D, Clarke M, Ghersi D, Liberati A, Petticrew M, et al. Preferred reporting items for systematic review and meta-analysis protocols (PRISMA-P) 2015: elaboration and explanation. Bmj. 2015;350:g7647.
25. Porritt K, Pearson A. The historical emergence of qualitative synthesis. Australia: Lippincott Williams & Wilkins; 2013.
26. Hannes K, Lockwood C. Pragmatism as the philosophical foundation for the Joanna Briggs meta-aggregative approach to qualitative evidence synthesis. J Adv Nurs. 2011;67(7):1632-42.
27. Lockwood C, Pearson A. A comparison of meta-aggregation and meta-ethnography as qualitative review methods. New York: Lippincott Williams & Wilkins; 2013.
28. Lockwood C, Porritt K, Munn Z, Rittenmeyer L, Salmond S, Bjerrum M, et al. Chapter 2: Systematic reviews of qualitative evidence. In: Aromataris E, Munn Z, editors. JBI Manual for Evidence Synthesis: JBI; 2020.
29. Lockwood C, Munn Z, Porritt K. Qualitative research synthesis: methodological guidance for systematic reviewers utilizing meta-aggregation. Int J Evid Based Healthc. 2015;13(3):179-87.
30. Tong A, Flemming K, McInnes E, Oliver S, Craig J. Enhancing transparency in reporting the synthesis of qualitative research: ENTREQ. BMC Med Res Methodol. 2012;12:181.

For peer review only

For peer review only - http://bmjopen.bmj.com/site/about/guidelines.xhtml

Ovid MEDLINE(R) and Epub Ahead of Print, In-Process, In-Data-Review & Other Non-Indexed Citations, Daily and Versions January 1, 2012 to November 10, 2022
11/11/2022

**SEARCH TERMS**			**RESULTS**
**Pain**			
	1	Pain/ or Acute Pain/ or Pain, Procedural/ or Pain Measurement/ or Pain Management/ or Pain Threshold/	
	2	(pain* or ache* or aching or distress* or suffer* or itch* or discomfort* or anxious or anguished or agony or agonising or anxiety).ab,kf,ti.	
	3	1 or 2	
**Removal of dressings**			
	4	Bandages/ or Bandages, Hydrocolloid/ or Occlusive Dressings/ or Adhesives/	
	5	Device Removal/	
	6	(remov* or redress* or chang* or select* or application* or cho?s* or apply* or "device deficienc*" or "adverse event*").ab,kf,ti.	
	7	5 or 6	
	8	4 and 7	
	9	((fastener* or adhesive* or tape* or taping or bandaid* or bandag* or dressing* or mucilage* or "sticky past*" or gum or latex or adherent* or adhering or seal*) adj4 (remov* or redress* or chang* or select* or application* or cho?s* or apply* or "device deficienc*" or "adverse event*")).ab,kf,ti.	
**Combined Sets**			
	10	8 or 9	
	11	3 and 10	
	12	limit 11 to (yr="2012 -Current" and (danish or dutch or english or norwegian or swedish))	1,703

For peer review only

Reporting checklist for protocol of a systematic review and meta-analysis.

Based on the PRISMA-P guidelines.

Title: "Patients' experiences with the application of medical adhesives to the skin: a qualitative systematic review protocol"

Section and Topic		Reporting Item	Page Number and Notes
**Title**			
Identification	#1a	Identify the report as a protocol of a systematic review	Title
Update	#1b	If the protocol is for an update of a previous systematic review, identify as such	n/a: This is not an update of a previous systematic review.
**Registration**			
	#2	If registered, provide the name of the registry (such as PROSPERO) and registration number	n/a: The protocol has yet to be submitted to PROSPERO.
**Authors**			
Contact	#3a	Provide name, institutional affiliation, e-mail address of all protocol authors; provide physical mailing address of corresponding author	1
Contribution	#3b	Describe contributions of protocol authors and identify the guarantor of the review	7
**Amendments**			
	#4	If the protocol represents an amendment of a previously completed or published protocol, identify as such and list changes; otherwise, state plan for documenting important protocol amendments	n/a

For peer review only

**Support**			
Sources	#5a	Indicate sources of financial or other support for the review	7
Sponsor	#5b	Provide name for the review funder and / or sponsor	7
Role of sponsor or funder	#5c	Describe roles of funder(s), sponsor(s), and / or institution(s), if any, in developing the protocol	7
**Introduction**			
Rationale	#6	Describe the rationale for the review in the context of what is already known	3
Objectives	#7	Provide an explicit statement of the question(s) the review will address with reference to participants, interventions, comparators, and outcomes (PICO)	4
**Methods**			
Eligibility criteria	#8	Specify the study characteristics (such as PICO, study design, setting, time frame) and report characteristics (such as years considered, language, publication status) to be used as criteria for eligibility for the review	4
Information sources	#9	Describe all intended information sources (such as electronic databases, contact with study authors, trial registers or other grey literature sources) with planned dates of coverage	4-5
Search strategy	#10	Present draft of search strategy to be used for at least one electronic database, including planned limits, such that it could be repeated	Supplemental Material
Study records - data management	#11a	Describe the mechanism(s) that will be used to manage records and data throughout the review	5

For peer review only

Study records - selection process	#11b	State the process that will be used for selecting studies (such as two independent reviewers) through each phase of the review (that is, screening, eligibility and inclusion in meta-analysis)	5
Study records - data collection process	#11c	Describe planned method of extracting data from reports (such as piloting forms, done independently, in duplicate), any processes for obtaining and confirming data from investigators	5
Data items	#12	List and define all variables for which data will be sought (such as PICO items, funding sources), any pre-planned data assumptions and simplifications	5
Outcomes and prioritization	#13	List and define all outcomes for which data will be sought, including prioritization of main and additional outcomes, with rationale	n/a: As this review only includes qualitative data, no outcomes can be defined. The phenomena of interest will be sought for.
Risk of bias in individual studies	#14	Describe anticipated methods for assessing risk of bias of individual studies, including whether this will be done at the outcome or study level, or both; state how this information will be used in data synthesis	5: As this study focusses on qualitative data, the methodological quality of the included studies will be assessed, not the risk of bias.
Data synthesis	#15a	Describe criteria under which study data will be quantitatively synthesised	n/a: As this study focusses on qualitative data, no quantitative data synthesis can take place.
Data synthesis	#15b	If data are appropriate for quantitative synthesis, describe planned summary measures, methods of handling data and methods of combining data from studies, including any planned exploration of consistency (such as I2, Kendall's τ)	n/a: As this study focusses on qualitative data, no quantitative data synthesis can take place.
Data synthesis	#15c	Describe any proposed additional analyses (such as sensitivity or subgroup analyses, meta-regression)	n/a: As this study focusses on qualitative data, no subgroup analysis or meta-regression can take place.

For peer review only

Data synthesis	#15d	If quantitative synthesis is not appropriate, describe the type of summary planned	6
Meta-bias(es)	#16	Specify any planned assessment of meta-bias(es) (such as publication bias across studies, selective reporting within studies)	n/a
Confidence in cumulative evidence	#17	Describe how the strength of the body of evidence will be assessed (such as GRADE)	n/a

This checklist was completed on 1. June 2023 using https://www.goodreports.org/, a tool made by the EQUATOR Network in collaboration with Penelope.ai

For peer review only
